# Atlas-based finite element analyses with simpler constitutive models predict personalized progression of knee osteoarthritis: data from the osteoarthritis initiative

**DOI:** 10.1038/s41598-023-35832-y

**Published:** 2023-06-01

**Authors:** Mika E. Mononen, Alexander Paz, Mimmi K. Liukkonen, Mikael J. Turunen

**Affiliations:** 1 Department of Technical Physics, University of Eastern Finland, Kuopio, Finland; 2 Escuela de Ingeniería Civil y Geomática, Universidad del Valle, Cali, Colombia; 3 Department of Clinical Radiology, Kuopio University Hospital, Kuopio, Finland

**Keywords:** Computational biophysics, Cartilage, Osteoarthritis, Mechanical properties, Predictive medicine

## Abstract

New technologies are required to support a radical shift towards preventive healthcare. Here we focus on evaluating the possibility of finite element (FE) analysis-aided prevention of knee osteoarthritis (OA), a disease that affects 100 million citizens in the US and EU and this number is estimated to increase drastically. Current clinical methods to diagnose or predict joint health status relies on symptoms and tissue failures obtained from clinical imaging. In a joint with no detectable injuries, the diagnosis of the future health of the knee can be assumed to be very subjective. Quantitative approaches are therefore needed to assess the personalized risk for the onset and development of knee OA. FE analysis utilizing an atlas-based modeling approach has shown a preliminary capability for simulating subject-specific cartilage mechanical responses. However, it has been verified with a very limited subject number. Thus, the aim of this study is to verify the real capability of the atlas-based approach to simulate cartilage degeneration utilizing different material descriptions for cartilage. A fibril reinforced poroviscoelastic (FRPVE) material formulation was considered as state-of-the-art material behavior, since it has been preliminary validated against real clinical follow-up data. Simulated mechanical tissue responses and predicted cartilage degenerations within knee joint with FRPVE material were compared against simpler constitutive models for cartilage. The capability of the atlas-based modeling to offer a feasible approach with quantitative evaluation for the risk for the OA development (healthy vs osteoarthritic knee, *p* < 0.01, AUC ~ 0.7) was verified with 214 knees. Furthermore, the results suggest that accuracy for simulation of cartilage degeneration with simpler material models is similar to models using FPRVE materials if the material parameters are chosen properly.

## Introduction

Osteoarthritis (OA) is the most common degenerative joint disease of the musculoskeletal system. It affects approximately 100 million citizens in the US and EU and this number is estimated to increase by 25% during the next 20 years due to the aging of the population^1,2^. Currently, there is no cure for OA and current clinical practice in OA diagnosis is not able to detect early tissue changes due to OA. Therefore, knee OA often progresses inevitably to a stage where an expensive total knee replacement (TKR) surgery costing up to 50 000€ is the only recommended clinical solution. It has been reported that over 600 000 TKR procedures are performed each year in the US among patients aged 50–59^3^. This generates annually over 30-billion-dollar direct costs to the US's economy, highlighting the need for effective preventative interventions for clinical practice. For a solution to be clinically applicable, it has to be scalable, robust, and accurate.

Recently, OA research has increasingly started focusing on the development of different approaches and methods based on machine learning (ML) algorithms^4–7^ and finite element analysis (FEA)^8–10^ to classify subjects at high risk for knee OA development. Especially, the aim has been to identify the high-risk subjects before any degenerative signs are detected from a clinical image. In ML approaches^4–7^, the prediction is based on the parameters that can be easily quantified, such as age, height, weight, parameters that are evaluated from clinical image, such as Kellgren–Lawrence (KL) grade or tibiofemoral angle, and parameters that are reported by the subject itself such as different physical activity levels and pain indexes. This generates a huge set of parameters that needs to be defined before making an accurate prediction. As some of the parameters are considerable subjective, such as pain and activity levels^11,12^, it may generate biases to the data that is utilized to train ML algorithms. Furthermore, some of the parameters that are needed for ML approaches may be too time consuming or unpractical to collect (such as biomarkers of blood or urine) from the subjects which may limit its usability as a clinical tool^13^.

In approaches based on predictions generated from finite element analysis^8–10^, the mechanical response of the cartilage tissue within the joint is simulated and combined with degenerative algorithms that include a mechanical threshold(s) beyond which tissue degeneration takes place. Similarly, as with ML approaches, FEA approaches have its own limitations to be utilized as a part of clinical evaluation. For instance, constitutive models for cartilage tissue that has been utilized to predict tissue degradation due to OA progression are experimentally validated only with a complex fibril-reinforced poroviscoelastic material (FRPVE) formulations^14–18^. As the FRPVE material can be considered to simulate mechanical tissue responses adequately (contact pressure, stress, strains, and cartilage degeneration) during various loading conditions (stress-relaxation) in articular cartilage, it can be considered as a reference material model. However, the main limitation is that implementation of the FRPVE material description is time consuming, needs extensive expertise to implement in different tissue shapes, and requires considerably more computational time compared to simpler material formulations. The second limitation in the current FEA workflow is related to the time that is needed in generation of a subject specific model geometry. This can be tackled with an atlas-based approach^8^, where the existing atlas is scaled based on the measured anatomical knee joint dimensions. However, currently the applicability of the atlas-based approach has been verified with very limited subject number.

The aim of this study is to verify the real capability of the atlas-based approach to predict the progression of knee OA utilizing different material formulations for cartilage. We hypothesized that mechanical parameters in simpler constitutive models for cartilage can be adjusted so that tissue mechanical response corresponds with those simulated with the complex FRPVE material description. This will promote the usability of simpler constitutive models in simulating tissue mechanical responses, and especially, when predicting personalized progression of knee OA. We also hypothesized that simpler constitutive models combined with atlas-based modeling enable clinically feasible solutions to help clinicians to target conservative interventions for patients who are at high risk for the development of knee OA.

## Materials and methods

### Workflow

**Figure 1:**
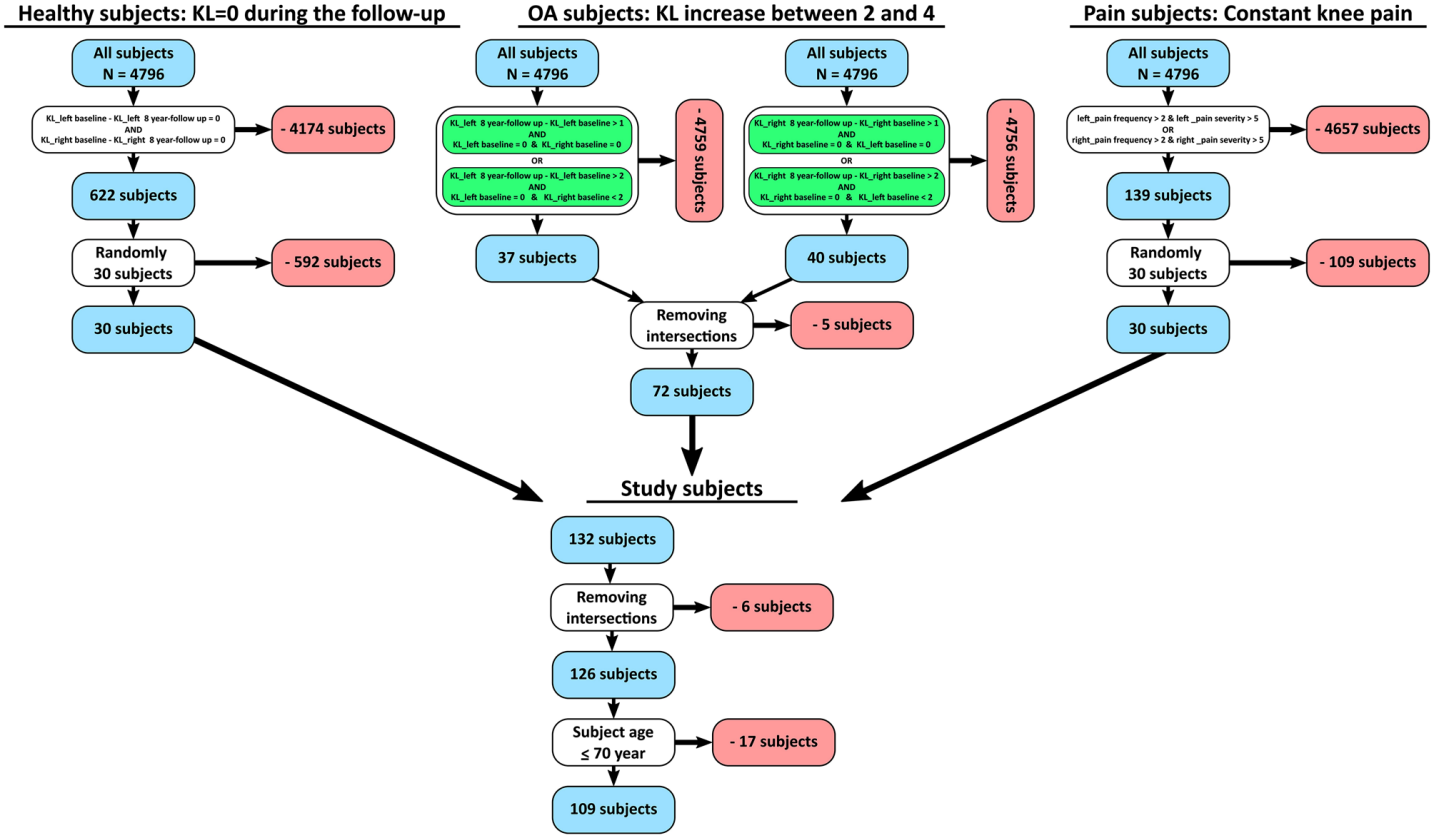
Inclusion criteria for the subject selection from the OAI database. In the selection of subjects with constant knee pain, pain frequency (V00P7LKFR (left knee) and V00P7RKFR (right knee) parameters from the OAI database) > 2 means daily knee pain to constant knee pain and pain severity (V00P7LKRCV (left knee) and V00P7RKRCV (right knee) parameters from the OAI database) > 5 means self reported pain level from 0 to 10, where 0 equals to “no pain” and 10 equals to “pain as bad as you can image”.

Magnetic resonance images (MRI) for 214 knee joints from 109 subjects were obtained from the osteoarthritis initiative database (OAI- https://nda.nih.gov/oai/). The study subjects were divided into three subject groups based on the 8-year follow-up Kellgren–Lawrence (KL) grade. Following inclusion criteria were used: *Healthy subjects—*30 random subjects whose KL grade at both knees remained zero during 8-year follow-up; *OA subjects—*KL grade increase at least by 2 in one knee so that the baseline KL in both knees was zero, *or*, KL grade increase at least by 3 in one knee so that its baseline KL grade was zero, whereas other knee’s baseline KL was restricted to be less than 2; *Pain subjects—*30 random subjects who had constant knee pain at the baseline in their left, right, or both knees. Then, intersections and patients older than 70 years were removed (Fig. 1). There were 222 knees having KL = 0, 24 having KL = 1 and 2 knees having KL = 2 in baseline. Finally, each knee joint was grouped based on their 8-year follow-up KL grade to three KL groups: (1) KL01: KL grades 0 and 1, (2) KL2: KL grade2, and (3) KL34: KL grades 3 and 4 (Table 1). Ethical approval for collecting all subject information was provided by the OAI. Knee MRI’s were carried out in accordance with FDA guidelines, whereas knee radiographs (x-ray) were carried out in accordance with typical guidelines for annual and total radiation dosage to research subjects. Written consent was obtained from all subjects prior to each clinic visit. The OAI study was approved by Institutional Review Board (IRB) for the University of California, San Francisco (UCSF) and its affiliates. The IRB approval was also obtained from all the four clinical sites located at Brown University in Rhode Island, Ohio State University in Columbus, Ohio, University of Maryland/Johns Hopkins University joint center in Baltimore, Maryland, and at the University of Pittsburgh in Pennsylvania. Further details related to the OAI data are available in the OAI web-site (https://nda.nih.gov/oai/).

**Table 1:** Variation in subject characteristics between different KL groups based on the KL grade at the 8-year follow-up. Number in parentheses indicates the number of pain subjects in the group.

	AGE [years]	BMI [kg/m^2^]	Medial cartilage thickness [mm]	Lateral cartilage thickness [mm]
Group Left knee				
KL01 N = 70 (15)	56.1 ± 6.2	28.3 ± 5.2	5.0 ± 1.0	5.6 ± 0.8
KL2 N = 20 (1)	58.1 ± 5.1	27.4 ± 4.7	4.9 ± 0.6	5.4 ± 0.8
KL34 N = 16 (2)	57.1 ± 6.2	31.8 ± 5.1	5.1 ± 1.0	5.3 ± 1.3
Group Right knee				
KL01 N = 69 (12)	56.0 ± 5.8	28.5 ± 5.6	5.1 ± 0.9	5.6 ± 1.0
KL2 N = 29 (4)	57.9 ± 6.1	29.0 ± 5.1	5.0 ± 1.0	5.6 ± 0.9
KL34 N = 10 (0)	56.0 ± 7.2	30.5 ± 5.9	5.3 ± 0.7	5.3 ± 0.6

Previously developed atlas-based FE approach^8^, utilizing the experimentally validated FRPVE constitutive model for cartilage, was considered as a reference model to simulate contact pressure, tissue tensile stress, compressive strain and cartilage degeneration under gait loading conditions. A transversely isotropic poroelastic material (TIPE) model from previous study^19^ and an optimized material model utilizing a homogeneous transversely isotropic poroelastic material (HTIPE) formulation were selected for comparison of simulated mechanical responses and predicted cartilage degeneration against to FRPVE constitutive model (Table 2). The optimization for the HTIPE material was conducted manually to achieve similar tissue deformations and tensile stresses as obtained with the FRPVE material utilizing a simplified tibiofemoral geometry. Finally, to test the capability of the simpler constitutive models for cartilage (HTIPE and TIPE) to simulate different tissue mechanical responses in a knee joint level model, simulated contact pressures, contact areas, pore pressure, and tissue tensile stresses and strains were compared against FRPVE constitutive model as a function of gait loading utilizing previously developed atlas-based modeling workflow^8^. All simulation were run in Abaqus (Dassault Systèmes). The FRPVE material implementation was done with UMAT subroutine that allows use of user-defined material behavior, whereas the simpler materials were implemented with inbuilt Abaqus options using the transverse isotropic material implementation (engineering constants). Details of the main equations and collagen fibril implementation of the FRPVE material, and convergence criteria for the joint level models are given in the “Supplementary material”.

**Table 2:** Material parameters of the FRPVE, TIPE and HTIPE material models for the femoral and tibial cartilages.

FRPVE material model^8^	Femoral cartilage	Tibial cartilage	
Collagen fibril network architecture	Depth-wise arcade-like	Depth-wise arcade-like	
${E}_{\upvarepsilon }$ (MPa)	150	23.06	
*E*_*0*_ (MPa)	0.92	0.18	
${E}_{\text{m}}$ (MPa)	0.215	0.106	
${\upsilon }_{\text{m}}$	0.15	0.15	
*η* (MPa s)	1062	1062	
${k}_{0}$ (× 10^−15^ m^4^N^−1^ s^−1^)	6	18	
*n*_f_*	0.8–0.15 Hz	0.8–0.15 Hz	

HTIPE material model	Femoral cartilage	Tibial cartilage	
Orientation of the plane of isotropy**	Parallel to surface	Parallel to surface	
${E}_{11}={E}_{22}$ (MPa)	60	50	
${E}_{33}$ (MPa)	3	3	
${v}_{12}$	0.42	0.42	
${v}_{13}={v}_{23}$	1.9	1.9	
${G}_{12}$(MPa)	5.25	4.4	
${G}_{13}={G}_{23}$ (MPa)	7.9	6.4	
*k* (× 10^−15^ m^4^N^−1^ s^−1^)	6	18	
*n*_*f*_	0.8	0.8	

TIPE material model^19^***	Superficial zone	Middle zone	Deep zone
Orientation of the plane of isotropy**	Parallel to surface	Parallel to surface	Parallel to surface
${E}_{11}={E}_{22}$ (MPa)	24	16.97	8.485
${E}_{33}$ (MPa)	0.46	0.46	0.46
${v}_{12}$	0.42	0.42	0.42
${v}_{13}={v}_{23}$	3	3	2.2
${G}_{12}$ (MPa)	8.45	5.98	2.99
${G}_{13}={G}_{23}$ (MPa)	12	8.45	4.24
*k * (× 10^−15^ m^4^N^−1^ s^−1^)	1	1	1
*n*_*f*_	0.8	0.8	0.8

*FRPVE material parameters:* ${E}_{\upvarepsilon }$ = the strain-dependent fibril network modulus, ${E}_{0}$ = the initial fibril network modulus, ${E}_{\text{m}}$ = the non-fibrillar matrix modulus,$v$_m_ = the Poisson’s ratio of the non-fibrillar matrix, *η* = the damping coefficient, ${k}_{0}$  = the initial permeability and *n*_*f*_ = the fluid fraction. *HTIPE and TIPE material parameters****:*** ${\text{E}}_{11}={\text{E}}_{22}=$  the in-plane Young’s modulus (representing primary collagen fibril orientation), ${\text{E}}_{33}$ = axial Young’s modulus (perpendicular to in-plane direction), ${v}_{ij}$ = the Poisson’s ratio that characterize the transverse strain in the *j*-direction, when the tissue is stresses in the *i*-direction., ${\text{G}}_{\text{ij}}$  = the shear modulus that characterize strain in i-plane in j-direction, k = the permeability and *n*_*f*_ = the fluid fraction.

* In FRPVE hz represents normalized depth of the tissue from cartilage surface (hz = 1) to cartilage bone interface (hz = 0).

** Orientation of the plane of isotropy represents primary collagen fibril orientation in cartilage.

*** In TIPE material model, both the femoral and tibial cartilages were considered to have identical depth-wise material parameters.

### Optimization of the HTIPE material

In the previously developed and verified algorithm to simulate cartilage degeneration^8^, tissue degeneration was based on the simulation of exceeded levels of tissue tensile stresses experienced by the collagen fibril network, utilizing the FRPVE constitutive model^17,18^. Thus, when optimizing the HTIPE material, the priority was to simulate identical tissue response in terms of tissue tensile stresses and cartilage deformation compared with the FRPVE constitutive model. It was assumed that similar stresses and deformations would produce similar distributions in other parameters (contact pressure, contact area, pore pressure, tensile strain).

**Figure 2:**
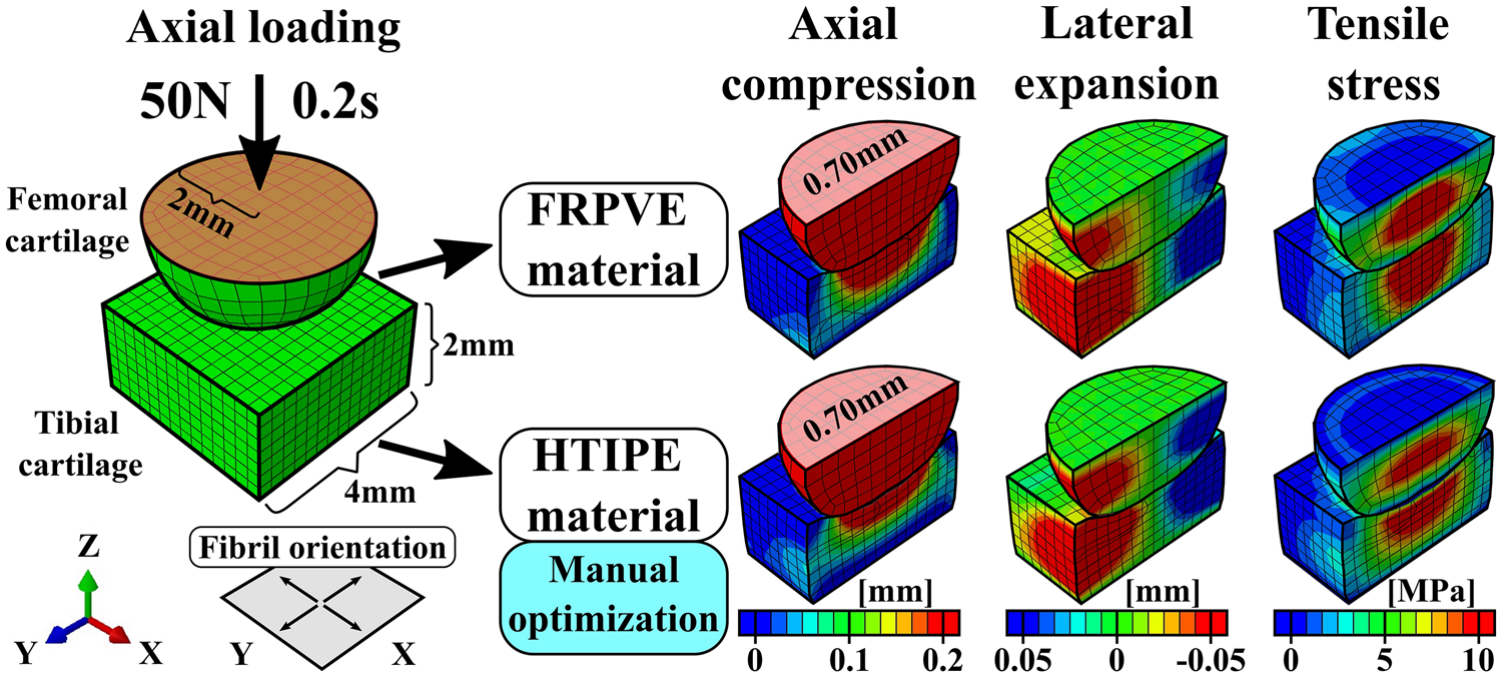
Final outcome (simulated mechanical responses) of the optimization of the mechanical parameters of the HTIPE material model using a simplified joint geometry for 0.2 s under an axial ramp load of 50 N. Fibril orientation refers to the collagen fibril structure implemented in the FRPVE model.

First, tibiofemoral joint was modeled as a *simplified joint geometry* utilizing a cuboid of 32 mm^3^ (2 mm × 4 mm × 4 mm) as tibial cartilage and a hemisphere with a radius of 2 mm as femoral cartilage (Fig. 2-left). The FRPVE material was implemented into the simplified joint geometry so that primary fibril orientations (4 primary fibrils^17,18^) were parallel to the femoral and tibial cartilages surface through whole tissue depth. In the HTIPE material model, material orientations were matched with the FRPVE material so that the xy-plane of the cartesian coordinate system, that defines material orientations, was according to primary fibril orientations in the FRPVE material.

After generation of the simplified joint geometry, the FRPVE material properties mimicking tibial and femoral cartilage were applied to the model geometry (Table 2). To match joint loading conditions during loading response, axial ramp loading of 50N was applied within 0.2 s (approximate time for loading response) on the top surface of femoral cartilage, while tibial bottom was fixed. Other degrees of freedom in femoral cartilage were kept fixed, except axial displacement that was controlled by the loading boundary condition. Biphasic contact was not used between femoral and tibial cartilage, and free fluid flow was not allowed at the free edges (Fig. 2-left). The outcomes of qualitative analysis on tissue tensile stresses and tissue deformations were used as references for optimizing material parameters for the HTIPE model.

As all material parameters are linked with the tissue’s mechanical response (stress level and deformation), some assumptions were made before manual optimization:

1. Void ratio and permeabilities are matched with FRPVE material (they were kept constant).
2. Initial Young’s moduli, Poisson’s ratios and shear moduli were based on the previous study^19^.
3. Due to primary fibril architecture in the FRPVE model (Fig. 2-left), Young’s moduli in the x and y direction (xy -plane) are considered to be even.

We were aware that mechanical responses in the FRPVE constitutive model are highly nonlinear with respect to local strain rates, due to the viscoelastic nature of the model, and thus it is impossible to get an identical match with simpler, non-viscoelastic materials. However, after about 100 iterations, both quantitative and qualitative comparisons between FRPVE and HTIPE material responses showed only minor differences between simulated stresses and tissue deformation. This was considered to justify further utilization of optimized HTIPE material (Fig. 2-right).

### Atlas-based method

**Figure 3:**
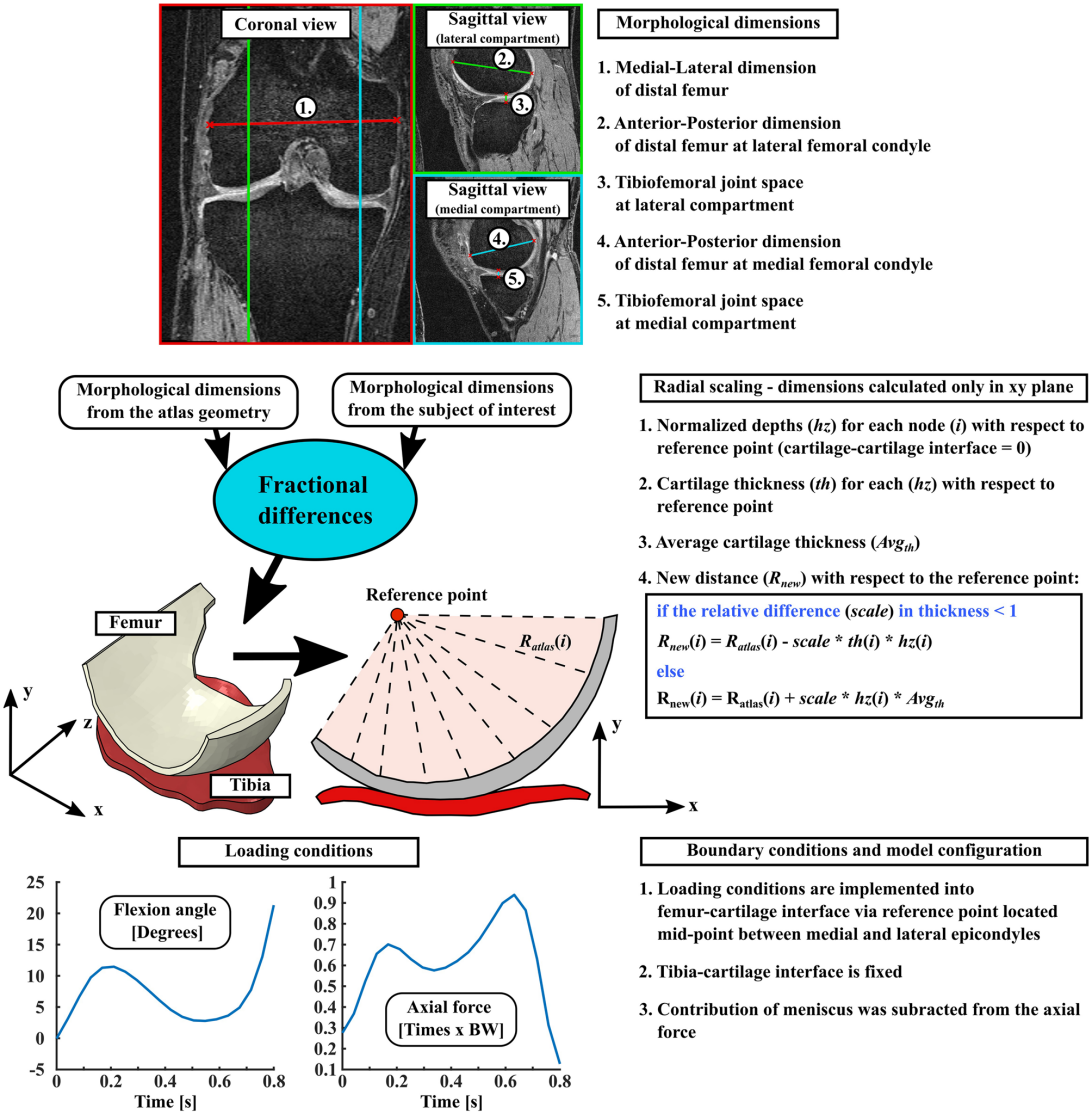
Overview of how the existing atlas FE geometry is scaled against measured morphological dimensions of subject of interest and how boundary conditions are implemented in the FE models considering body weight of subject of interest. The justification and how meniscus subraction is performed are explained in detail in a previous study^8^.

Subject specific FE models for each knee joint were generated utilizing the atlas-based approach^8^. Shortly, an existing atlas geometry (includes FE geometry and data from the morphological dimensions of distal femur and tibiofemoral joint) is scaled based on the relative differences in morphological dimensions of distal femur and tibiofemoral joint space between the subject of interest and atlas geometry (Fig. 3-top). Unlike as reported in the original atlas-based approach^8^, cartesian cartilage thickness scaling was replaced with a radial scaling as it minimizes geometrical distortion in cases, when there exist substantial differences in cartilage thicknesses between the subject of interest and atlas geometry (Fig. 3-middle). A generic gait loading condition was scaled based on the body weight of the subject of interest and implemented into the FE models, similarly as in the original atlas-based approach paper (Fig. 3-bottom)^8^.

### Simulation of cartilage degeneration

Cartilage degeneration was simulated as a function of ageing using age-dependent thresholds for tissue failure^20^. Thus, the following equations were used to determine the age-dependent threshold values (${T}_{{\upsigma }_{\text{f}}}$) for tensile stresses to initiate of cartilage degeneration^8^:

1 $$T_{{\upsigma }_{\text{f}}} = 30 {\text{ MPa}},\quad {\text{if}}\;\;({\text{Age}} < 30),$$

2 $$T_{{\upsigma }_{\text{f}}} = 30{\text{ MPa}} - \left( {\left( {{\text{Age}} - 30} \right)\left( {20/15} \right)} \right){ ,}\quad {\text{if}}\;\; \left( {{3}0 \, \le {\text{ Age }} \le { 45}} \right),$$

3 $$T_{{\upsigma }_{\text{f}}} = 10\; {\text{MPa}} - \left( {\left( {{\text{Age}} - 45} \right)\left( {3/20} \right)} \right), \quad {\text{if}} \;\;(45 \, < {\text{Age}} \le 65),$$

4 $$T_{{\upsigma }_{\text{f}}} = 7{\text{ MPa}} - \left( {\left( {{\text{Age}} - 65} \right)\left( {2/100} \right)} \right){, }\quad {\text{if}} \;\;({65 } < {\text{Age}} \le 75),$$

5 $$T_{{\text{f}}} = 6.8{\text{ MPa},}\quad {\text{if}}\;\;({\text{Age}} > 75).$$

Based on the given age of subject (b = baseline age) and the desired simulated OA progression time (8y = simulated time + b), volumetric cartilage degeneration (DEG(Age)) was calculated for each time point as the sum of the volumes of the elements where the threshold (${T}_{{\upsigma }_{\text{f}}}$) was exceeded (any given time point during the gait loading). Thus, the simulated progression of degeneration (D) after 8-year follow-up can be formulated as follows:

6 $${\text{D}} = {\text{DEG}}\left( {{\text{8y}}} \right) - {\text{ DEG}}\left( {\text{b}} \right).$$

The progression of degeneration was calculated using post-processing in Matlab (Mathworks) after simulating the tensile stresses during gait loading.

### Statistical analysis

To emphasize the effect of geometry scaling, average differences in different mechanical parameters between FRPVE and simpler constitutive models were presented in the relation with thickness scaling. Correlations for each parameter was evaluated by the Pearson’s correlation. Bland–Altman plots were created and analyzed for agreement between the mechanical responses between FRPVE and the simpler material models. Cartilage degenerations were simulated with each constitutive material, and ROC (receiver operating characteristic) curves were calculated to demonstrate suitability of simpler constitutive models to predict progression of knee OA. Non-parametric Mann–Whitney U-test (two independent samples test) was used to evaluate group-wise (KL grade at the 8-year follow-up) statistical differences in the predicted degenerations within each constitutive models and between pain and no pain subjects, whereas non-parametric Wilcoxon signed rank test (two related samples test) was used to evaluate statistical differences within each KL group (KL grade at the 8-year follow-up) between different constitutive models. In all statistical tests p < 0.05 was considered as the level of significance.

## Results

**Figure 4:**
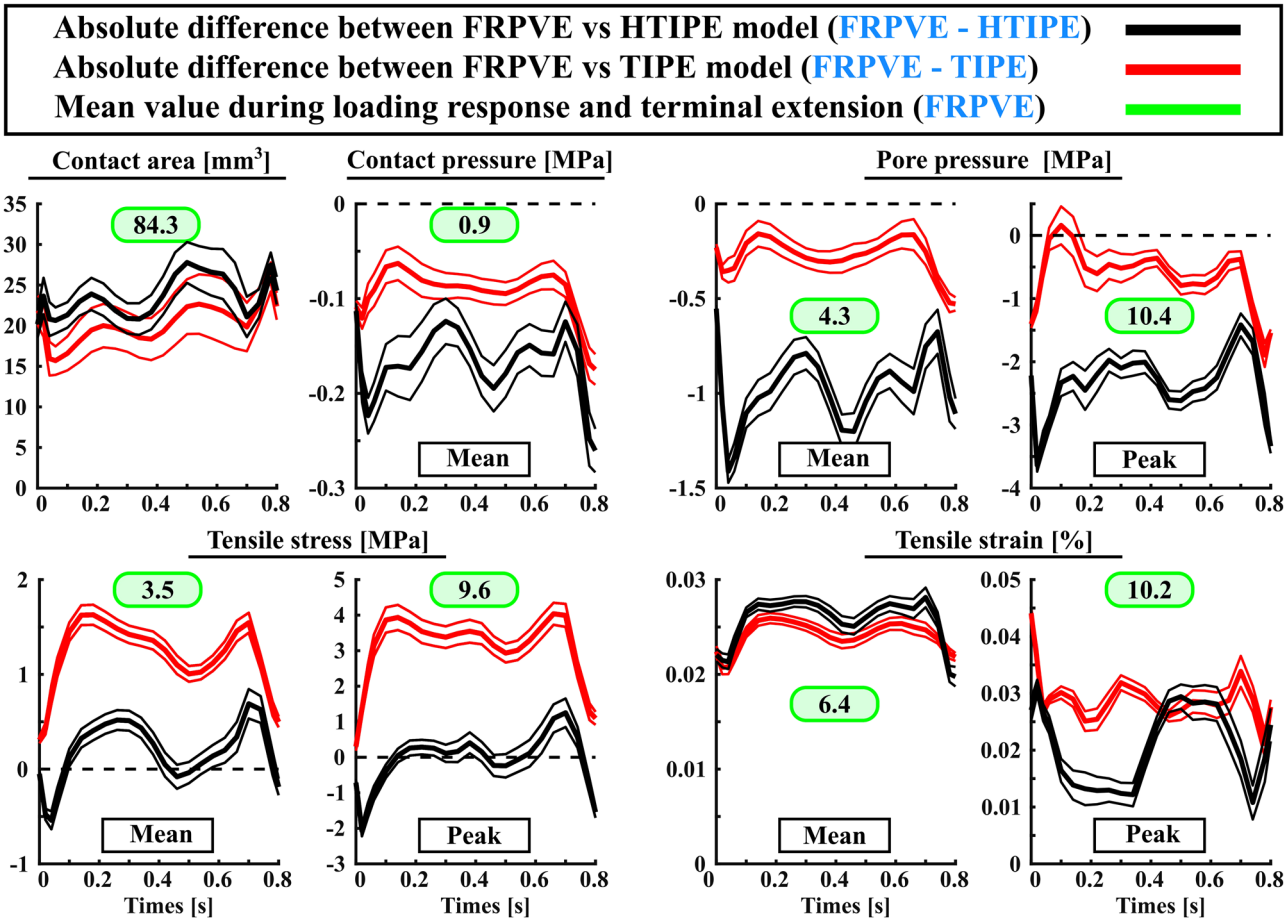
Simulated absolute differences (thick lines) in mechanical responses between FRPVE and simpler models (HTIPE and TIPE) with 95% confidence interval (thin lines). The “Mean” curves are calculated as average over the contact area and the ”Peak” curves are the peak maxima over the contact area as a function of stance phase. The green box indicates the average calculated over peak values generated during the loading response and terminal extension. This helps to interpret the simulated differences, i.e., are the simulated differences high or low.

**Figure 5:**
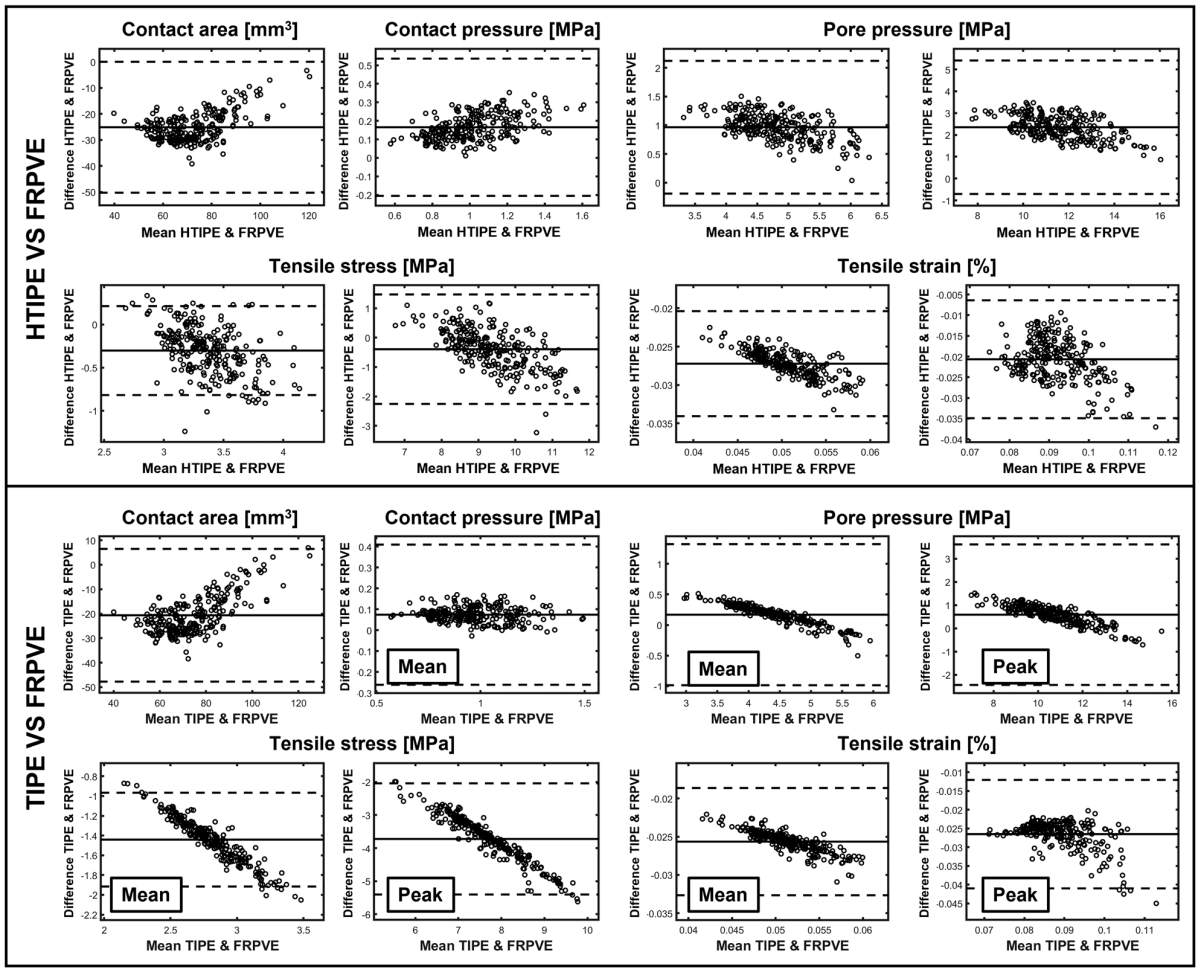
Bland–Altman plots of mechanical responses between FRPVE and HTIPE, and FRPVE and TIPE models. The values are averages calculated over peak values generated during loading response and terminal extension during the gait cycle (as in Fig. 4 green boxes). The mean difference with ± 1.96 SD is indicated with the dashed lines.

Simpler constitutive models for femoral and tibial cartilages were unable to replicate all mechanical responses obtained with the FRPVE constitutive model. In general, the simpler constitutive models either underestimated (contact area, tensile stress, tensile strain) or overestimated (contact pressure, pore pressure) the mechanical responses (Figs. 4 and 5). However, the HTIPE model was able to replicate simulated tensile stresses (mean and peak values) with adequate accuracy compared to the FRPVE model (average difference < 10%). The average difference in other parameters (mean and peak values) was > 20%. TIPE model was unable to replicate tensile stresses compared to the FRPVE model (average difference > 30%), but the average differences in contact area and contact pressure were < 20% and the average difference in pore pressure (mean and peak values) was < 15% compared to the FRPVE model. Interestingly, both simpler models were unable to replicate tensile strains from the FRPVE model (average difference > 20%).

**Figure 6:**
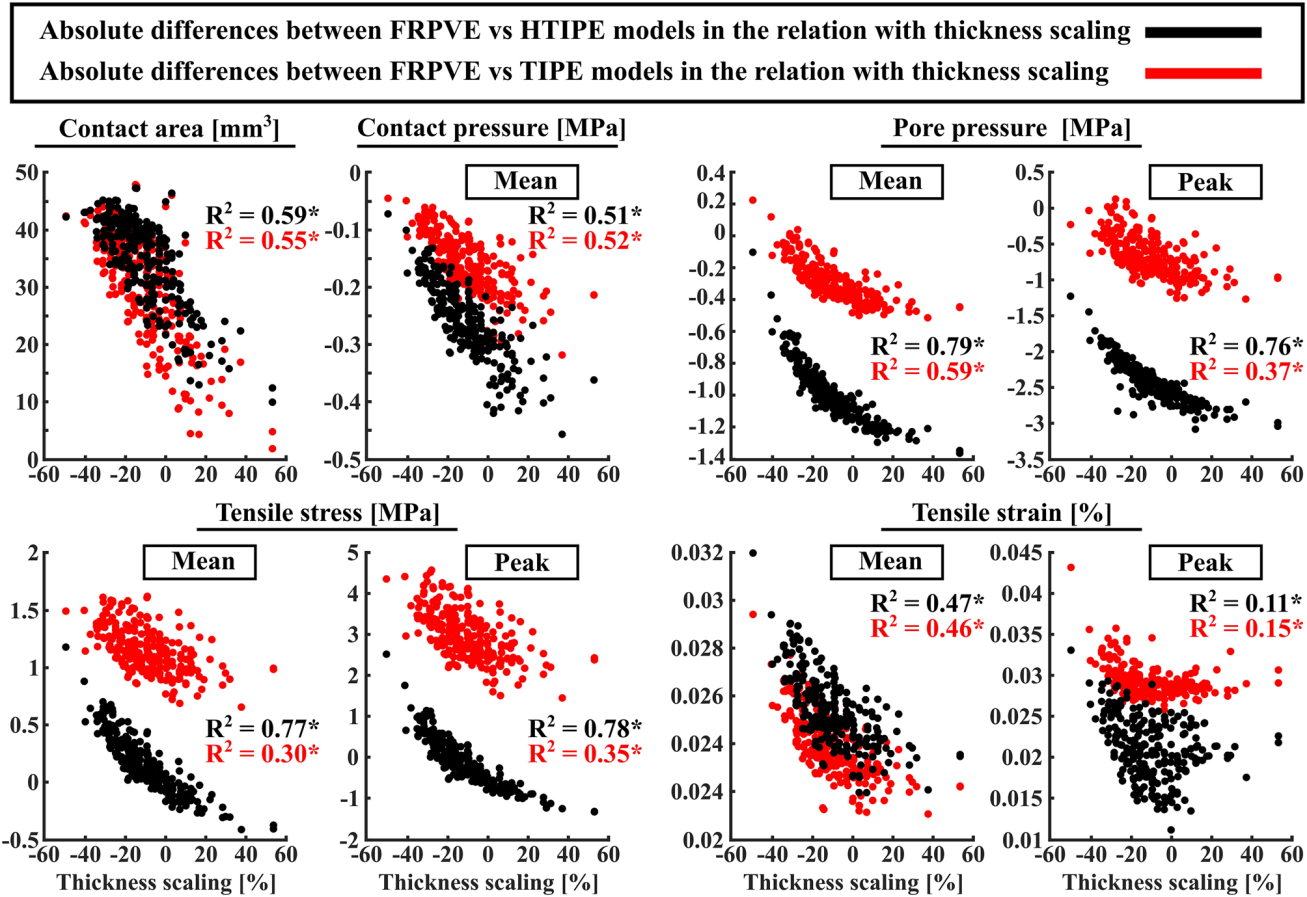
Simulated absolute differences in mechanical responses between FRPVE and simpler models (HTIPE and TIPE) in the relation with thickness scaling. A single point indicates the average difference through the entire stance phase for one knee model. R^2^ indicates the Pearson’s correlation against to cartilage thickness scaling, *p < 0.01.

The cartilage thickness scaling (atlas scaling) significantly affected the differences in the mechanical responses from the TIPE and HTIPE models, as seen in the correlations between cartilage thickness scaling factor and FRPVE-HTIPE and FRPVE-TIPE mechanical responses (*p* < 0.01 for all, Fig. 6).

**Figure 7:**
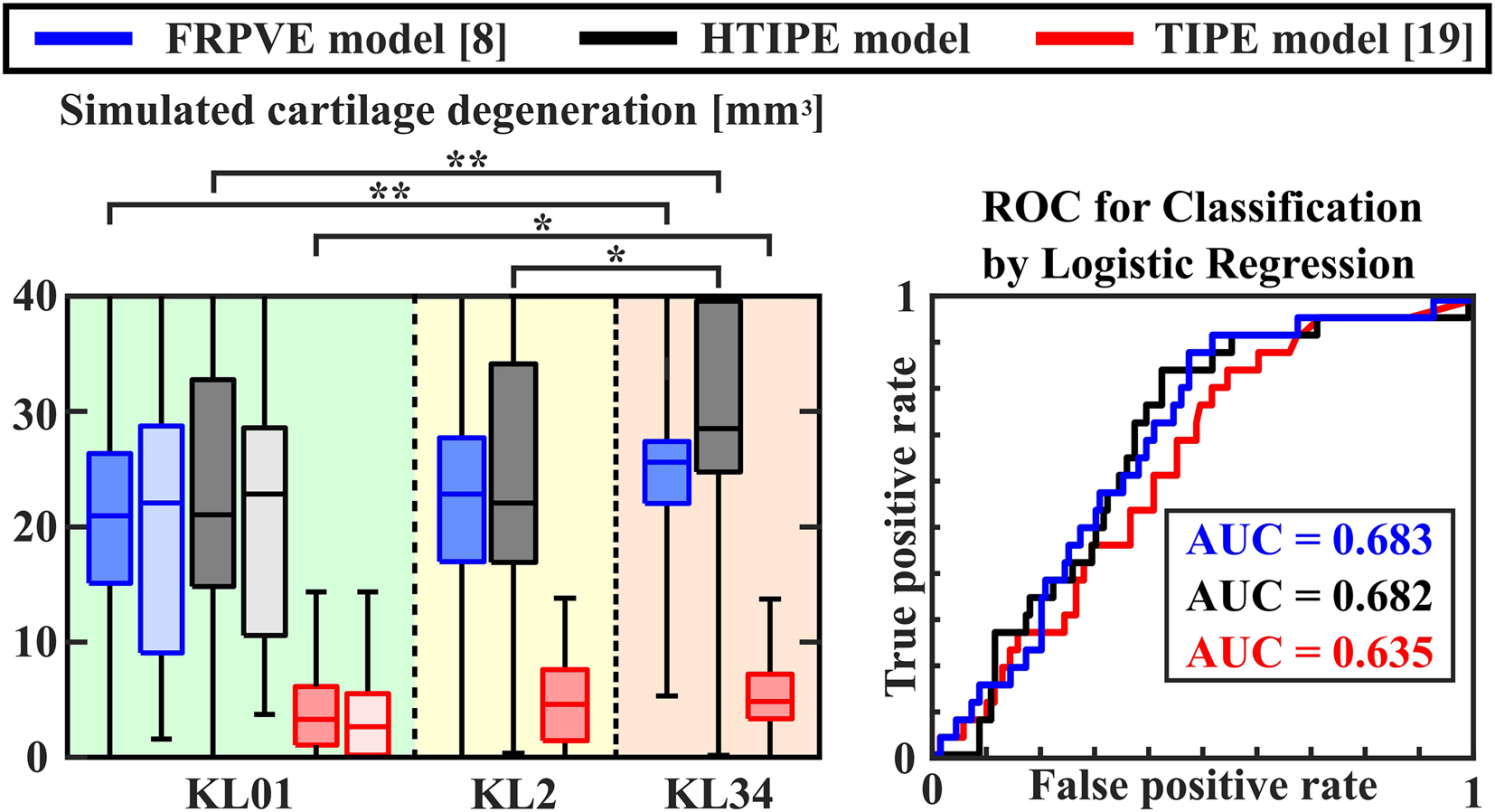
Simulated cartilage degenerations with all models and resulted ROC (receiver operating characteristic) curves with AUC (area under the ROC curve) values indicating classification superiority. Mann–Whitney U-test *p < 0.05 and **p < 0.01. Green, yellow and orange background indicate KL01, KL2 and KL34 groups, respectively. Box plot with light color in KL01 group indicates knee joints from the subjects having knee pain (see Fig. 1 for inclusion criteria for those).

The range of simulated cartilage degenerations in all KL groups were similar in FRPVE and HTIPE models but were underestimated in TIPE model (Fig. 7-left). Wilcoxon signed rank test showed statistical differences (*p* < 0.01) between each model (FRPVE vs TIPE, FRPVE vs HTIPE, TIPE vs HTIPE) in each KL group. Based on the Mann–Whitney U-test, all models showed significantly larger cartilage degeneration in the KL34 group compared to the KL01 group (*p* < 0.01 for FRPVE and HTIPE, and *p* < 0.05 for TIPE). Additionally, the HTIPE model showed significant difference also between KL2 and KL34 groups (*p* < 0.05). There was no difference between cartilage degeneration in pain subjects and all subjects in any model.

The classification capability between KL01 and KL34 groups were in a similar range in the FRPVE and HTIPE models (AUC = 0.683 and 0.682, respectively), whereas the TIPE model showed the poorest classification capability (AUC = 0.635) (Fig. 7-right). The simulated cartilage degenerations between FRPVE and HTIPE, and FRPVE and TIPE models correlated significantly (*p* < 0.01) in each KL group (Table 3).

**Table 3:** Pearson correlations (R^2^) of simulated cartilage degenerations between FRPVE and HTIPE, and FRPVE and TIPE models in each KL group.

Group	FRPVE vs HTIPE	FRPVE vs TIPE
KL0-1	0.792**	0.707**
KL2	0.738**	0.810**
KL3-4	0.587**	0.570**
Pooled KL0-4	0.771**	0.719**

**p < 0.01.

## Discussion

In the current study, the mechanical responses of cartilage were simulated within various knee joints under generic gait loading with the HTIPE material model, where material parameters were optimized against to the mechanical responses simulated in experimentally validated FRPVE material model. The previously used TIPE material model^19^ was only used to understand the potential limitations of predicting mechanical responses or cartilage degeneration with material models with different stiffness (the softness of TIPE model was not known in advance). Although the optimized HTIPE material model produced nearly identical mechanical responses to the FRPVE material model using a simple joint geometry, it was concluded that only tissue tensile stresses were reproduced with sufficient accuracy compared to the FRPVE knee joint model responses. Furthermore, it was concluded that the variation in cartilage thickness in the knee joint model alters substantially the simulated differences in the mechanical responses between the material models. However, this had only small contribution to predicted cartilage degeneration between the FRPVE and HTIPE models, since the simulated excessive joint loads are considered to be the main mechanisms behind the initiation of OA development and progression^21–24^. Therefore, when classifying subjects into the KL01 and KL34 groups, the classification accuracy (moderate) was similar in both material models.

The material parameters of the TIPE model were derived from a previous study^19^. Shortly, it was optimized to a fibril-reinforced poroelastic (FRPE) model without the viscous response of the collagen fibril network. Thus, it is not surprising that the simulated mechanical responses were constantly higher or lower as obtained from the FRPVE material model. In contrast, the HTIPE model was optimized for the mechanical response obtained from the FRPVE material model, and in the simplified joint geometry, primary aim of optimization (tensile stress and cartilage deformation) was reached. However, in knee joint geometry, only tensile stresses were moderately reproduced compared to the mechanical responses of the FRPVE material model. This can be explained mainly by the joint shape and cartilage thickness variation at the tibiofemoral contact during gait that generates varying load/strain rates on the cartilage surface between the models. As the FRPVE material is highly sensitive to load/strain-rate, due to viscoelasticity of the model, this causes mechanical responses that are not linearly dependent on load magnitude (this also occurs experimental measurements^25,26^). For this reason, simulated differences between FRPVE and HTIPE, and FRPVE and TIPE models were not constant, even though the mechanical responses in FRPVE and HTIPE models were the same in the simplified joint geometry using the linear strain rate.

The extent to which the thickness of the existing atlas model was scaled (when the patient specific models were generated) significantly affected the differences in the simulated mechanical responses between FRPVE vs HTIPE, and FRPVE vs TIPE material models. In cases where cartilage thickness of the existing atlas was only slightly scaled (< 20%), the average mechanical responses for tissue tensile stresses were in good agreement between FRPVE and HTIPE material models (difference < 1 MPa). Other simulated parameters were either overestimated or underestimated. However, importantly the thickness scaling has a (almost) linear response between the simulated mechanical responses of the different models (FRPVE vs HTIPE; FRPVE vs TIPE). This indicates that it is possible to estimate the mechanical response of the FRPVE model from these simple models, knowing how a change in geometry affects the differences in the simulated responses, as simulated in this work. This is important as complex FRPVE material models are computationally heavy compared to simpler material models. For instance, in the current study, simulation of a FRPVE model took ~ 2 h, whereas simulation of an HTIPE/TIPE model took ~ 20 min.

When simulating cartilage degeneration, the FRPVE and HTIPE models performed similarly, while the TIPE model underestimated the degeneration. The poorer performance in TIPE model is explained by the age-dependent threshold for initiation of cartilage degeneration that works better in materials that produce higher tensile stresses. Although the AUC value was only moderate to classify subjects between KL01 (who will remain healthy) and KL34 (who will have OA) groups, it must be considered that average BMI’s of the different groups were between overweight and obesity (27-32 g/cm^2^). In other words, in terms of baseline BMI, most of the subjects in each group had already a high risk for the onset and development of knee OA. When this information is reflected to the obtained ROC values, the prediction accuracy seems very promising. Interestingly, how the patients felt current knee pain had no effect on simulated degenerations. This indicates that knee pain is not associated with morphological changes in knee shape or cartilage thickness. This does not exclude the possibility that the pain is caused by a knee injury that has not yet been diagnosed.

Various machine learning (ML) models have been developed for classification of high and low risk subjects^4–6^. In these ML models, AUC values ranged from 0.6 to 0.8. However, it should be noted that some of those models require up to 112 predictor variables to make the classification. Collecting of such number of predictors is not clinically feasible timewise. Furthermore, it should be acknowledged that ML models are not capable of simulating quantitatively effects of different interventions such as weight loss or gait retraining that is possible with FEA based simulations, as utilized in the current study. However, it is possible to combine ML models with FEA-based simulations into a single tool^27^. This might be the next route for classification algorithms generation for evaluating subject specific risks for the onset and progression of knee OA.

Despite the encouraging results on the personalized risk of onset and progression of knee OA, there are some limitations to this study. First, only medial compartment of the knee was utilized in the simulation. Although OA usually initiates on the medial compartment, some patients exhibit the initiation on the lateral compartment^28,29^. Consideration of this aspect might offer an improvement in the classification results (Fig. 7). We consider this as critical limitation that should be addressed in coming papers. Second, loading conditions were assumed to be identical for all subjects. It is well known that loading conditions vary among different subjects^30,31^. We utilized a generic loading that was scaled based on the body weights of subjects^8,32^. This can be considered as a feasible simplification since wrong estimation for personalized loading condition might produce even higher inaccuracies compared with the generic loading conditions. Third, when biomechanical models are optimized, they may become overfitted to their calibration conditions. Depending on the complexity of the model, even a small change in the boundary condition or geometry can affect its accuracy. In this work, this phenomenon was clearly observed in how the tibiofemoral cartilage thickness affected the differences in the simulated mechanical responses in the HTIPE and TIPE models compared to the simulated mechanical responses in the FRPVE model. This should be considered when using the results of this work in future studies. Fourth, the inflammatory mechanisms were not considered in our predictive model for OA progression. It is known that during the early stages of post-traumatic OA due to joint injury, pro-inflammatory cytokines derived from the synoviocytes of the synovial lining are secreted to the synovial fluid and then diffuse and advect into the cartilage, reducing biosynthesis and predisposing to tissue degeneration^33^. However, it should be noted that it is extremely challenging to validate this mechanism with whole knee joint level models, as suitable multi-year follow-up data with are currently not available. Last, material properties were not subject specific and quantitative measures that indicate cartilage health such as T2 values from MRI were not utilized^9^. In future studies, current joint integrity should be addressed when making predictive models. In the current study, an existing degeneration algorithm^8^ with high subject number was utilized.

The presented results suggest that simpler material models are capable to predict subject specific progression of knee OA if material parameters are selected properly. In the future, the presented workflow with simpler material model should be tested against to larger cohort data with a wider range of subject characteristics. The contribution of the lateral compartment should also be taken into account when making predictions for the onset and progression of knee OA. Furthermore, FEA based simulations merged with ML models could provide an accurate and fast clinical tool for prediction of osteoarthritis and simulation of different conservative preventative interventions, such as weight loss and gait retraining.

## Supplementary Information

Supplementary Information 1.

## Data Availability

The data created and analysed during the current study are available from the corresponding author upon reasonable request.

## References

[1] Hootman JM, Helmick CG, Barbour KE, Theis KA, Boring MA (2016). Updated projected prevalence of self-reported doctor-diagnosed arthritis and arthritis-attributable activity limitation among US Adults, 2015–2040. Arthritis Rheumatol..

[2] Barbour KE, Helmick CG, Boring M, Brady TJ (2017). Vital signs: prevalence of doctor-diagnosed arthritis and arthritis-attributable activity limitation—United States, 2013–2015. MMWR Morb. Mortal Wkly. Rep..

[3] Kremers HM (2014). Prevalence of total hip and knee replacement in the United States. J. Bone Jt. Surg. Am..

[4] Joseph GB, McCulloch CE, Nevitt MC, Link TM, Sohn JH (2022). Machine learning to predict incident radiographic knee osteoarthritis over 8 Years using combined MR imaging features, demographics, and clinical factors: data from the Osteoarthritis Initiative. Osteoarthr. Cartil..

[5] Tiulpin A (2019). Multimodal machine learning-based knee osteoarthritis progression prediction from plain radiographs and clinical data. Sci. Rep..

[6] Yoo HJ, Jeong HW, Kim SW, Kim M, Lee JI, Lee YS (2022). Prediction of progression rate and fate of osteoarthritis: Comparison of machine learning algorithms. J. Orthop. Res..

[7] Guan B (2020). Deep learning approach to predict pain progression in knee osteoarthritis. Osteoarthr. Cartil..

[8] Mononen ME, Liukkonen MK, Korhonen RK (2019). Utilizing atlas-based modeling to predict knee joint cartilage degeneration: data from the osteoarthritis initiative. Ann. Biomed. Eng..

[9] Lampen, N., Su, H., Chan, D. D. & Yan, P. T_2_ Mapping refined finite element modeling to predict knee osteoarthritis progression. In *2021 43rd Annual International Conference of the IEEE Engineering in Medicine & Biology Society (EMBC)*, 4592–4595 (2021). 10.1109/EMBC46164.2021.9629780. 10.1109/EMBC46164.2021.9629780 34892238

[10] Bolcos PO (2022). Subject-specific biomechanical analysis to estimate locations susceptible to osteoarthritis—Finite element modeling and MRI follow-up of ACL reconstructed patients. J. Orthop. Res..

[11] Younger J, McCue R, Mackey S (2009). Pain outcomes: A brief review of instruments and techniques. Curr. Pain Headache Rep..

[12] van Weering M, Vollenbroek-Hutten M, Hermens H (2011). The relationship between objectively and subjectively measured activity levels in people with chronic low back pain. Clin. Rehabil..

[13] Bonakdari H, Jamshidi A, Pelletier J-P, Abram F, Tardif G, Martel-Pelletier J (2021). A warning machine learning algorithm for early knee osteoarthritis structural progressor patient screening. Ther. Adv. Musculoskelet. Dis..

[14] Julkunen P, Kiviranta P, Wilson W, Jurvelin JS, Korhonen RK (2007). Characterization of articular cartilage by combining microscopic analysis with a fibril-reinforced finite-element model. J. Biomech..

[15] Ebrahimi M (2019). Elastic, viscoelastic and fibril-reinforced poroelastic material properties of healthy and osteoarthritic human tibial cartilage. Ann. Biomed. Eng..

[16] Mäkelä JTA, Huttu MRJ, Korhonen RK (2012). Structure–function relationships in osteoarthritic human hip joint articular cartilage. Osteoarthr. Cartil..

[17] Wilson W, van Donkelaar CC, van Rietbergen B, Ito K, Huiskes R (2004). Stresses in the local collagen network of articular cartilage: a poroviscoelastic fibril-reinforced finite element study. J. Biomech..

[18] Wilson W, van Donkelaar CC, van Rietbergen B, Ito K, Huiskes R (2005). Erratum to ‘Stresses in the local collagen network of articular cartilage: a poroviscoelastic fibril-reinforced finite element study’ [Journal of Biomechanics 37 (2004) 357–366] and ‘A fibril-reinforced poroviscoelastic swelling model for articular cartilage’ [Journal of Biomechanics 38 (2005) 1195–1204]. J. Biomech..

[19] Klets O, Mononen ME, Tanska P, Nieminen MT, Korhonen RK, Saarakkala S (2016). Comparison of different material models of articular cartilage in 3D computational modeling of the knee: Data from the Osteoarthritis Initiative (OAI). J. Biomech..

[20] Kempson GE (1982). Relationship between the tensile properties of articular cartilage from the human knee and age. Ann. Rheum. Dis..

[21] Radin EL, Paul IL, Pollock D (1970). Animal joint behaviour under excessive loading. Nature.

[22] Miller RH, Edwards WB, Brandon SCE, Morton AM, Deluzio KJ (2014). Why don’t most runners get knee osteoarthritis? A case for per-unit-distance Loads. Med. Sci. Sports Exerc..

[23] Horisberger M, Fortuna R, Valderrabano V, Herzog W (2013). Long-term repetitive mechanical loading of the knee joint by in vivo muscle stimulation accelerates cartilage degeneration and increases chondrocyte death in a rabbit model. Clin. Biomech..

[24] Seedhom BB (2006). Conditioning of cartilage during normal activities is an important factor in the development of osteoarthritis. Rheumatology.

[25] Rodriguez ML, Li L (2017). Compression-rate-dependent nonlinear mechanics of normal and impaired porcine knee joints. BMC Musculoskelet. Disord..

[26] Charlebois M, McKee MD, Buschmann MD (2004). Nonlinear tensile properties of bovine articular cartilage and their variation with age and depth. J. Biomech. Eng..

[27] Paz A, Orozco GA, Korhonen RK, García JJ, Mononen ME (2021). Expediting finite element analyses for subject-specific studies of knee osteoarthritis: a literature review. Appl. Sci..

[28] Wise BL (2012). Patterns of compartment involvement in tibiofemoral osteoarthritis in men and women and in whites and African Americans. Arthritis Care Res. (Hoboken).

[29] Baliunas AJ (2002). Increased knee joint loads during walking are present in subjects with knee osteoarthritis. Osteoarthr. Cartil..

[30] Kumar D, Manal KT, Rudolph KS (2013). Knee joint loading during gait in healthy controls and individuals with knee osteoarthritis. Osteoarthr. Cartil..

[31] Schwachmeyer V, Kutzner I, Bornschein J, Bender A, Dymke J, Bergmann G (2015). Medial and lateral foot loading and its effect on knee joint loading. Clin. Biomech..

[32] Bergmann G (2014). Standardized loads acting in knee implants. PLoS ONE.

[33] Wojdasiewicz P (2019). The role of inflammatory and anti-inflammatory cytokines in the pathogenesis of osteoarthritis. Mediat Inflamm..

