# Supplementary material for: Atlas-based finite element analyses with simpler constitutive models predict personalized progression of knee osteoarthritis: data from the osteoarthritis initiative
Source: Sci Rep. 2023 Jun 1;13:8888. doi: 10.1038/s41598-023-35832-y (PMC10235100; doi:10.1038/s41598-023-35832-y)
Supplement: Supplementary file 1 — Supplementary Information 1. [file 41598_2023_35832_MOESM1_ESM.docx]

**Supplementary material:**

**Atlas-based finite element analyses with simpler constitutive models predict personalized progression of knee osteoarthritis: Data from the Osteoarthritis Initiative**

Mika E. Mononen^1^, Alexander Paz Carvajal^1,2^, Mimmi K. Liukkonen^3^, Mikael J. Turunen^1^

^1^Department of Technical Physics, University of Eastern Finland, Kuopio, Finland

^2^Escuela de Ingeniería Civil y Geomática, Universidad del Valle, Cali, Colombia

^3^Department of Clinical Radiology, Kuopio University Hospital, Kuopio, Finland

# Material and Methods

**Jacobian matrix (FRPVE model):**

In FRPVE material, the Jacobian ${}^{4}\boldsymbol{C}$ matrix was defined as follows (Wilson, 2005):

${}^{4}\boldsymbol{C}\boldsymbol{=}\frac{\boldsymbol{1}}{\boldsymbol{J}} \left( \boldsymbol{\sigma}\frac{\delta J}{\delta\boldsymbol{F}}\boldsymbol{+ J}\frac{\delta\boldsymbol{\sigma}}{\delta\boldsymbol{F}} \right)^{\boldsymbol{RT}}\cdot\boldsymbol{F}^{T}$**,** (S.1)

where $\boldsymbol{J}$ is the determinant of the deformation gradient tensor $\boldsymbol{F}$, $\boldsymbol{\sigma}$ is the Cauchy stress, and $\delta$is the size of time increment.

**Update of stress tensor (FRPVE model):**

At each integration point the total stress in the solid matrix is given by the sum of the

stresses in the non-fibrillar matrix and the sum of all fibril stresses by:

$\boldsymbol{\sigma}_{tot=}\boldsymbol{\sigma}_{nf}+ \boldsymbol{\sigma}_{f,global}$ (S.2)

where $\boldsymbol{\sigma}_{nf}$and $\boldsymbol{\sigma}_{\boldsymbol{f,global}}^{\boldsymbol{i}}$ are the stresses in the non-fibrillar matrix and in each individual fibril, both with respect to the global coordinate system. Since the non-fibrillar matrix was assumed to be linear elastic and isotropic, the non-fibrillar matrix stresses can be computed using Hooke’s Law. To determine the fibril stresses at each integration point, the initial orientation of each fibril is given by a unit vector $\nu_{0}$. After deformation the new unit fibril vector $\nu_{new}$ can be calculated as follows:

$\vec{\nu}_{new}= \frac{\boldsymbol{F}\cdot\nu_{0}}{\left\| \boldsymbol{F}\cdot\nu_{0} \right\|}$ , (S.3)

The logarithmic fibril strain can be computed as:

$\varepsilon_{f}=log\left\| \boldsymbol{F}\cdot\nu_{0} \right\|$. (S.4)

This strain is then used to calculate the fibril stress $\sigma_{f}$ that can be described as follows:

| $\sigma_{f}= -\frac{\eta}{2\sqrt{{(\sigma}_{f}-E_{0}\varepsilon_{f})}E_{\varepsilon}}\dot{\sigma}_{f}+E_{0}\varepsilon_{f}+\left( \eta\mathbf{+} \frac{\eta E_{0}}{2\sqrt{{(\sigma}_{f}-E_{0}\varepsilon_{f})}E_{\varepsilon}} \right)\dot{\varepsilon}_{f}, \varepsilon_{f}\geq0$ | (S.5) |
| --- | --- |
| $\sigma_{f}=0, \varepsilon_{f}\boldsymbol{\leq}0$ |  |

where *E*_0_ and *E*_ε_ are the initial and the strain-dependent fibril network moduli, respectively, and $\dot{\sigma}_{f}$ and $\dot{\varepsilon}_{f}$ are the fibril stress- and strain-rates, respectively, and *η* and $\varepsilon_{f}$ are the damping coefficient and the fibril strain. In cartilage FRPVE material, we used 4 primary and 13 secondary fibrils for each integration point of the continuum element. Thus, the total fibril stress tensor is the sum of the stress tensors of individual fibrils:

| $\boldsymbol{\sigma}_{f,global}= \sum_{i = 1}^{4} \rho_{z}C\sigma_{f,i}\vec{\nu}_{new,i}\bigotimes\vec{\nu}_{new,i}+\sum_{j= 4}^{17} \rho_{z}\sigma_{f,j}\vec{\nu}_{new,j}\bigotimes\vec{\nu}_{new,j}$ | (S.6) |
| --- | --- |

where $\rho_{z}$ is the depth-dependent fibril density (which was assumed to be constant through tissue depth ($\rho_{z}=1$)), $C$ is the ratio between primary and secondary fibrils, $\vec{\nu}_{new,j}$ is the current fibril orientation vector of the *i*:th or j:th fibril and $\bigotimes$ denotes the outer product. With the total stress tensor, the stiffness tensor can now be computed through the variation in Kirchhoff stress as follows:

$\delta\left( J\boldsymbol{\sigma} \right)=J^{4}\boldsymbol{C}:\boldsymbol{D}_{\delta}$ (S.7)

Here, $\boldsymbol{D}_{\delta}$ is the virtual rate of deformation, defined as:

$\boldsymbol{D}_{\delta}=sym(\delta\boldsymbol{F} \cdot\boldsymbol{F}^{-1})$. (S.8)

For $\delta\left( J\boldsymbol{\sigma} \right)$ it holds that

$\delta\left( J\boldsymbol{\sigma} \right)= \delta J\boldsymbol{\sigma}$ + $J \delta\boldsymbol{\sigma}$ (S.9)

$= \boldsymbol{\sigma}\delta J$ + $J \delta\boldsymbol{\sigma}$ (S.10)

***=*** $\left( \boldsymbol{\sigma}\frac{\delta J}{\delta\boldsymbol{F}}\boldsymbol{+}J\frac{\delta\boldsymbol{\sigma}}{\delta\boldsymbol{F}} \right)\mathbf{:}\delta\boldsymbol{F}$ (S.11)

***=*** ${}^{\boldsymbol{4}}\boldsymbol{A}$***:*** $\delta\boldsymbol{F}$***.*** (S.12)

**Maximum permitted rate of change of pore pressure in each step and convergence criteria (joint level models):**

*Soils, consolidation, end=PERIOD, utol=1e+09, cetol=1e+09

*Controls, reset

*Controls, analysis=discontinuous

*Controls, parameters=time incrementation

10, 16, , , , , , , , ,

*Controls, parameters=constraints

, , , 0.1, 0.1, , , 1

*Controls, parameters=line search

10, , , ,

*Controls, parameters=field, field=displacement

1000., 1, , , , , ,

*Controls, parameters=field, field=hydrostatic fluid pressure

1000., 1, , , , , ,

*Controls, parameters=field, field=rotation

1000., 1., , , , , ,

*Controls, parameters=field, field=pore fluid pressure

1000., 1, , , , , ,

**References**

W. Wilson, “An explanation for the onset of mechanically induced cartilage damage,” Phd Thesis 1 (Research TU/e / Graduation TU/e), Biomedical Engineering], Technische Universiteit Eindhoven, DOI:10.6100/IR583122
